# A comparative assessment of dilution correction methods for spot urinary analyte concentrations in a UK population exposed to arsenic in drinking water

**DOI:** 10.1016/j.envint.2019.03.069

**Published:** 2019-09

**Authors:** Daniel R.S. Middleton, Michael J. Watts, David A. Polya

**Affiliations:** a Section of Environment and Radiation, International Agency for Research on Cancer (IARC), Lyon, France; b Inorganic Geochemistry, Centre for Environmental Geochemistry, British Geological Survey, Nottingham, UK; c School of Earth and Environmental Sciences & Williamson Research Centre for Molecular Environmental Science, University of Manchester, Manchester, UK

**Keywords:** As^IMM^, urinary inorganic As and methylated metabolites, CI, confidence interval, GM, geometric mean, HPLC, high performance liquid chromatography, ICP-MS, inductively coupled plasma mass spectrometry, ln, natural logarithm, QC, quality control, SG, specific gravity, UFR, urinary flow rate, Biomonitoring, Arsenic, Urine, Hydration correction

## Abstract

Spot urinary concentrations of environmental exposure biomarkers require correction for dilution. There is no consensus on the most appropriate method, with creatinine used by default despite lacking theoretical robustness. We comparatively assessed the efficacy of creatinine; specific gravity (SG); osmolality and modifications of all three for dilution correcting urinary arsenic. For 202 participants with urinary arsenic, creatinine, osmolality and SG measurements paired to drinking water As, we compared the performance corrections against two independent criteria: primarily, (A) correlations of corrected urinary As and the dilution measurements used to correct them - weak correlations indicating good performance and (B) correlations of corrected urinary As and drinking water As - strong correlations indicating good performance. More than a third of variation in spot urinary As concentrations was attributable to dilution. Conventional SG and osmolality correction removed significant dilution variation from As concentrations, whereas conventional creatinine over-corrected, and modifications of all three removed measurable dilution variation. Modified creatinine and both methods of SG and osmolality generated stronger correlations of urinary and drinking water As concentrations than conventional creatinine, which gave weaker correlations than uncorrected values. A disparity in optima between performance criteria was observed, with much smaller improvements possible for Criterion B relative to A. Conventional corrections – particularly creatinine - limit the utility spot urine samples, whereas a modified technique outlined here may allow substantial improvement and can be readily retrospectively applied to existing datasets. More studies are needed to optimize urinary dilution correction methods. Covariates of urinary dilution measurements still warrant consideration.

## Highlights

• There is no consensus on the most robust method for the urinary dilution correction of environmental chemicals.
• We assessed the efficacy of conventional and modified dilution correction methods on urinary arsenic concentrations.
• Conventional corrections – namely creatinine - limit the utility of spot urine. A modification may allow improvement.

## 1 Introduction

Urinary biomonitoring is a widely used tool for environmental exposure assessment, the evaluation of suspected carcinogens and toxins, exposomics and metabolomics, nutritional science and numerous other applications. Despite its longstanding employment, the non-invasiveness, logistical and analytical appeal of urinary biomonitoring are yet to be matched by robust data processing and interpretation protocols (Sobus et al., 2015; Barrett, 2015). Urinary analyte concentrations are subject to variation from several factors (Aylward et al., 2014): (i) sample collection timing relative to exposure; (ii) inter/intra-individual toxicokinetics and (iii) physicochemical characteristics of the urinary matrix. We address the third factor, namely the inter-individual hydration-driven dilution variation in spot urine samples, in the present paper. Failure to properly account for this and other sources of variation in urinary analyte concentrations is currently limiting the potential of a convenient and growing resource of invaluable biomonitoring data for environmental research and other epidemiological investigations (Middleton et al., 2016a).

When comparing urinary biomonitoring results between different individuals within a population, it is necessary to account for inter-individual differences in sample dilution so that ‘like with like’ comparisons can be made and robust relationships between biomarker concentrations and external exposure metrics and/or health endpoints can be explored. Several approaches exist for correcting urinary analyte concentrations for dilution variation, the most common of which are creatinine - whereby analyte concentrations are expressed as ratios against creatinine concentrations (Eq. (1)); specific gravity (SG) (Eq. (2)) (Levine, 1945) and osmolality correction (replacing SG-1 for urinary osmolality in Eq. (2)).

(1) $$C_{\mathrm{cor}}=C_{\mathrm{vol}}/C_{\mathrm{cr}},$$

where C_cor_ is dilution corrected analyte concentration (in the above case expressed as the ratio μg/g creatinine), C_vol_ is the measured, volume based analyte concentration (typically in μg/L) and C_cr_ is creatinine concentration in g/L.

(2) $$C_{\mathrm{cor}}=C_{\mathrm{vol}}\times \left(\left({\mathrm{SG}}_{\mathrm{ref}}-1\right)/\left({\mathrm{SG}}_{\mathrm{meas}}-1\right)\right),$$

where SG_ref_ is the reference value (e.g. often the median study group SG) to which analyte concentrations are normalised and SG_meas_ is that measured in a given specimen. We note that Eq. (1) is equivalent to substituting creatinine for SG-1 in Eq. (2) and normalizing to a creatinine concentration of 1 g/L.

These corrections utilise measurements that act as proxies for urinary flow rate (UFR) – the volume of urine produced in a given timeframe and the ultimate property of interest (Middleton et al., 2016a; Hays et al., 2015; Araki et al., 1990), albeit cumbersome to quantify. Creatinine correction assumes that creatinine – a breakdown product of muscle creatine phosphate - is excreted at a constant rate and varies only by UFR. This has been repeatedly disputed, with creatinine concentrations having been shown to vary by demographic group (Barr et al., 2005), protein intake (Mayersohn et al., 1983) and muscle mass (Baxmann et al., 2008). Osmolality, regarded as the definitive measure of urinary concentration (Leech and Penney, 1987), accounts for the entire solute content of the sample. Specific gravity is an estimate of osmolality conveniently measured by refractometry and, while close agreement has been demonstrated between the two measurements (Barber and Wallis, 1986), overestimations of SG relative to osmolality can occur in the presence of high molecular weight solutes (e.g. samples from subjects with albuminuria) (Leech and Penney, 1987). Osmolality too has been shown to vary by demographic and physical attributes, albeit less so than creatinine concentrations (Yeh et al., 2015).

Comparatively less attention has been paid to the mathematical modification of dilution corrections, which assume that both the analyte under investigation and dilution measurement change proportionally in response to UFR if performed in their conventional manner (Eqs. (1), (2)). This assumption was shown to be invalid almost 30 years ago by Araki et al. (1990), who reported analyte-specific, log-linear relationships between analyte concentrations and UFR derived from single individuals across various hydration states:

(3) $$\log\;C_{\mathrm{vol}}=a-b\;\log\;\mathrm{UFR},$$

where *a* and *b* are analyte-dependent, empirically determined regression coefficients. This finding may have particularly profound implications for creatinine correction, which is susceptible to the specific excretion kinetics of creatinine as opposed to osmolality and SG which reflect total solute content, unless correcting concentrations of an analyte with a slope (*b*) close to that of creatinine. It is, however, possible to account for these excretion differences in correction equations (Araki et al., 1990). We therefore propose for consideration a pre-existing modification of all three commonly used adjustment methods based on previous expansions of Araki's work (Vij and Howell, 1998a; Middleton et al., 2016b) as follows:

(4) $$C_{\mathrm{cor}}=C_{\mathrm{vol}}\times {\left(D_{\mathrm{ref}}/D_{\mathrm{meas}}\right)}^{z},$$

where *D* is the dilution measurement being used (cf. Eq. (2); minus unity in the case of SG) and *z,* in theory, is empirically derived by dividing the regression slope of As (*b*_*As*_) against UFR by that of the dilution metric (*b*_*D*_) against UFR, hence *z = b*_*As*_*/b*_*D*_.

As already noted, *D*_*ref*_ for creatinine is routinely = 1 g/L, therefore the modified equation can also be expressed as follows (cf. Eq. (1)):

(5) $$C_{\mathrm{cor}}=C_{\mathrm{vol}}/{C_{\mathrm{cr}}}^{z},$$

Despite evidence of its inefficacy (Middleton et al., 2016a; Nermell et al., 2008a), conventional creatinine correction remains a routine and often default approach to adjusting urinary analytes, as demonstrated by a Google Scholar search using the term “*urine;* (one of *creatinine, specific gravity, osmolality*) *correction OR adjustment*”, which returned 1980, 234 and 61 results, respectively and an almost identical proportional distribution when *arsenic* was added to the term. More studies are beginning to consider alternatives (Athanasiadou et al., 2018; Bulka et al., 2017), but there is a paucity of investigations using objective criteria to comparatively assess different approaches. We previously proposed four criteria for assessing the performance of dilution correction methods (Table 1). This set of criteria is by no means exhaustive and the suitability of each criterion may be study-specific; nevertheless it provides a good starting point for investigation.

**Table 1:** Suggested criteria for assessing performance of urinary biomonitoring dilution correction methods (Middleton et al., 2016a, Middleton et al., 2016b, Middleton et al., 2016c, Middleton et al., 2016d). The criteria employed in the present study are labelled A and B.

Criterion	Description	Performance metric
A	Correlation between corrected spot analyte concentrations and UFR (by proxy in the present paper).	Weaker correlations indicating good performance.
Not used in the present study	Correlation between corrected spot analyte concentrations and an independent measure of internal dose, e.g. analyte concentrations in blood.	Stronger correlations indicating good performance.
Not used in the present study	Correlation of spot analyte concentrations with analyte excretion over 24 h or composite 24 h concentrations.	Closer agreement/lower variation in spot samples indicating good performance.
B	Correlation of spot analyte concentrations with an independent measure of/proxy for external exposure e.g. drinking water analyte concentrations.	Stronger correlations indicating good performance.

In the present study, we aim to comparatively assess the performance of conventional creatinine, SG, osmolality and a modified application of all three methods in dilution correcting concentrations of urinary arsenic (As) in a population of UK participants exposed to various levels of As from private drinking water supplies. As the removal of hydration-driven dilution variation is the intended result of correction methods, we primarily assess performance in relation to Criterion A in Table 1, but also assess how adjustments affect the correlation between urinary and drinking water As concentrations (Criterion B in Table 1). Inorganic As is an established carcinogen (IARC, 2012) of the lung, bladder and skin and is associated with numerous other non-communicable diseases, including cardiovascular disease (Navas-Acien et al., 2005) and diabetes mellitus (Navas-Acien et al., 2008), for which urinary biomonitoring is the preferred measure of exposure assessment (Orloff et al., 2009). To our knowledge, this is the first investigation to assess the performance of these correction methods on a study population with paired urine and drinking water samples, thus allowing assessment against two independent criteria.

## 2 Materials and methods

### 2.1 Ethical approval

Ethical approval for the study was granted by the University of Manchester Research Ethics Committee (Ref 13068) and the NHS Health Research Authority National Research Ethics Committee (NRES) (Ref 13/EE/0234). In accordance with approved guidelines, written informed consent was obtained from all participants.

### 2.2 Participant recruitment and sample collection

Paired urine and drinking water samples were collected from participants' homes in Cornwall, south west England between November and December 2013 as part of an investigation into As exposure from various environmental sources (Middleton et al., 2016c; Middleton et al., 2016d; Middleton et al., 2017; Middleton et al., 2018). Point-of-use drinking water samples were collected from the tap most frequently used for consumption in LDPE containers (Nalgene, USA). Spot (85% first morning void), mid-stream urine samples collected and refrigerated by participants in 60 mL HDPE containers (Nalgene, USA) and stored in a cool box during transit. An electronic questionnaire was administered to obtain age and gender.

### 2.3 Chemical analysis

On arrival at the laboratory, SG was measured using a temperature-corrected, handheld PAL-10-S digital refractometer (Atago, Japan) prior to samples being filtered through 0.45 μm Acrodisc® syringe filters (PALL Life Sciences, USA) into 30 mL HDPE containers (Nalgene, USA) and frozen at −30 °C. Samples were thawed at room temperature before subsequent analyses were conducted. Creatinine concentrations were determined by the colorimetric Jaffe method (Jaffe, 1886) using a Randox liquid assay kit and a Randox RX Imola chemistry analyser. Osmolality was measured by cryoscopic (freezing point) osmometry using a Gonotect Osmomat 030 instrument (Gonetec, Germany). Total As in drinking water and urinary arsenic speciation were determined using previously detailed methodologies and QC procedures (Middleton et al., 2016d). In brief, inductively coupled plasma mass spectrometry (ICP-MS) (Agilent 7500 Series, Agilent Technologies, USA) was used to analyse drinking water samples and urinary arsenic speciation was performed with high performance liquid chromatography coupled to ICP-MS (HPLC-ICP-MS). Urinary inorganic As and methylated metabolites (As^IMM^) was calculated as the sum of inorganic As(III), inorganic As (V), methylarsonate (MA) and dimethylarsinate (DMA) species. This is the routine biomarker of As exposure; does not incorporate non-toxic arsenobetaine and is therefore more reflective of exposure to inorganic arsenic from drinking water.

### 2.4 Quality control

Accuracy was assessed using certified reference materials (CRM), which were analysed simultaneously with drinking water and urine samples: NIST SRM 1643e Trace Elements in Water (National Institute of Standards and Technology, USA) (certified value: 58.98 ± 0.70As μg/L, recovery: 100% (*n* = 4), precision: 3%) and NIES No.18 Human Urine (National Institute for Environmental Studies, Japan) (total As certified value: 137 ± 11 μg/L, recovery: 99% (*n* = 14), precision: 5%; AB certified value: 69 ± 12 μg/L, recovery: 92% (*n* = 18), precision: 5%; DMA certified value: 36 ± 9 μg/L, recovery: 115% (*n* = 18), precision: 12%). The accuracy of creatinine concentrations in urine was assessed with two different quality control samples which were analysed with each batch - Randox Acusera Assayed Urine Quality Control Level 2 (99% mean recovery, *n* = 4) and Level 3 (102% mean recovery, *n* = 4). (Background contamination was monitored using procedural blanks for urine and drinking water analyses, reagent (0.5% nitric acid) blanks for drinking water analysis and filter blanks for urine analysis. Precision was assessed by performing duplicate analyses. Duplicate measurements were made on 12% (*n* = 25) of urine samples for osmolality (1% mean percentage difference). To assess signal stability and the possibility of drift resulting from high urinary matrices, intra-batch duplicates were analysed for urinary speciation (mean percentage differences for detected species: AB: 2% (*n* = 5), MA: 2% (*n* = 1), DMA: 5% (*n* = 6)).

### 2.5 Urinary dilution corrections

Conventional creatinine and SG correction were performed as already described using Eq. (1) and Eq. (2), respectively and conventional osmolality correction was performed using Eq. (6).

(6) $$C_{\mathrm{cor}}=C_{\mathrm{vol}}\times \left({\mathrm{Osm}}_{\mathrm{ref}}/{\mathrm{Osm}}_{\mathrm{meas}}\right)$$

Values of 521 mOsm/kg and 1.016 were used for *Osm*_*ref*_ and *SG*_*ref*_ – the study group median osmolality and SG, respectively.

### 2.6 Correction performance assessment criteria

Dilution correction methods were assessed, primarily, against the aforementioned criterion (Table 1):

**Criterion A**: *Correlations between corrected spot analyte concentrations and UFR* - for the present paper, correlations between corrected As^IMM^ concentrations and the dilution measurement (creatinine, SG or osmolality) used to correct them were calculated. For the correction method to give the desired effect, **no correlation** should remain post-correction.

Performance in relation to the following independent criterion (Table 1) was additionally assessed:

**Criterion B**: *Correlation of spot analyte concentrations with an independent measure of/proxy for external exposure* e.g. *drinking water analyte concentrations* – for the present paper, correlations between drinking water total As and urinary As^IMM^ were calculated, with **stronger correlations** assumed to indicate better correction performance.

### 2.7 Modified dilution corrections

**Fig. 1:**
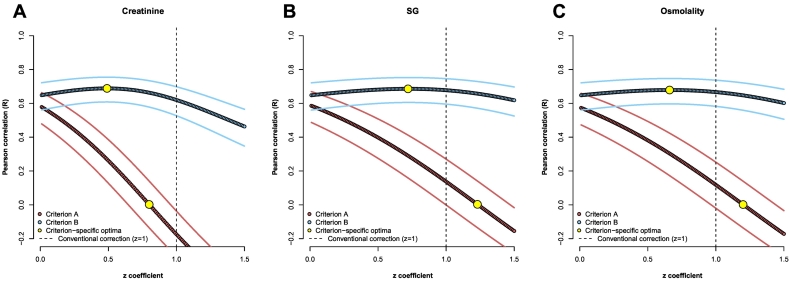
A graphical representation of the numerical derivation of the z coefficients used in the modified creatinine (A), SG (B) and osmolality (C) corrections. The optimum z coefficients for Criterion A (i.e. corresponding to R = 0 – lower yellow points) were selected. The 95% confidence intervals of Pearson correlations are represented by the upper and lower lines running parallel to points. (For interpretation of the references to colour in this figure legend, the reader is referred to the web version of this article.)

Modified dilution corrections were performed as per Eq. (4) (SG/osmolality) and Eq. (5) (creatinine). The *z* coefficient used in both equations was derived (Fig. 1) using a previous numerical approach (*z = b*_*As*_ / *b*_*D*_) (Vij and Howell, 1998b). Given that no published values for *z* were available, values between 0 and 1.5 at increments of 0.01 were imputed in a loop of correlation calculations between urinary As^IMM^ and the dilution measurement under modification (Criterion A). The *z* value yielding the optimal correlation (*R* = 0) was selected, resulting in *z* values of 0.80, 1.23 and 1.2 for creatinine, SG and osmolality, respectively. The limitations of this approach are addressed in the discussion.

### 2.8 Statistical analysis

Statistical analysis and graphics were generated using R version 3.4.3 (base package) and the RStudio GUI. Urinary analyte data were positively skewed, so geometric means (GM) were calculated. For this reason, Pearson correlations of urinary As^IMM^ concentrations against creatinine, SG, osmolality and drinking water As concentrations were calculated on natural log (ln) transformed data with significance tests (*p*-values) and 95% confidence intervals (CI). Linear regression models for trend lines were also performed on ln-transformed variables. To test the significance of the difference between correlations of, for example, drinking water total As and urinary As^IMM^ yielded by different correction methods, the Williams's test was performed using the psych package (these correlations are not independent from one another if they share a common variable - drinking water total As).

## 3 Results

### 3.1 Participant characteristics

Urinary As^IMM^, in addition to creatinine, osmolality and SG were measured in 202 urine samples, each of which had a paired concentration of total As in household drinking water. The mean age of participants, all of whom were of white European ethnicity, was 62, Demographic characteristics and urinary analyte summary statistics of the study group are shown in Table 2. Detailed urinary As speciation results and drinking water total As concentrations (GM: 1.0 μg/L; range: 0.01–233 μg/L) for this study group have been reported elsewhere (Middleton et al., 2016d).

**Table 2:** Characteristics of the study group (*n* = 202).

Demographic group	*n* (%)
Male	106 (52)
Female	96 (48)
18–39 years old	8 (4)
40–49 years old	26 (13)
50–59 years old	41 (20)
60–69 years old	72 (36)
≥70 years old	55 (27)


Urinary analytes	GM (range)
Creatinine (g/L)	0.83 (0.16–3.9)
SG	1.016 (1.005–1.031)
Osmolality (mOsm/kg)	520 (184–1161)
As^IMM^ (μg/L)	5.8 (0.9–124)

### 3.2 Effects of hydration corrections on urinary arsenic

Table 3 shows the effect of dilution corrections on urinary As^IMM^ concentrations – both statistical distributions and at an individual level. All correction methods reduced the geometric standard deviation among concentrations but, with the exception of both osmolality corrections, increased the range of concentrations. Relative to uncorrected concentrations, large differences in As^IMM^ concentrations for single individuals were observed between correction methods. In particular, conventional creatinine correction resulted in notably large differences (GM: 39%; max: 524%) relative to uncorrected concentrations. This was not the case for modified creatinine correction, which resulted in differences comparable to both osmolality and SG correction.

**Table 3:** Geometric means (GM), geometric standard deviations (GSD) and ranges of urinary As^IMM^ concentrations (μg/L except both creatinine methods - μg/g Cre) corrected by different methods and GMs and ranges of the intra-individual absolute percentage difference of urinary As^IMM^ concentrations corrected by different methods, relative to uncorrected concentrations.

Correction method	GM ± GSD (range) urinary As^IMM^	Absolute % difference (range) relative to uncorrected
Uncorrected	5.8 ± 2.3 (0.9–124)	
Creatinine (conventional)	6.9 ± 2.0 (1.8–177)	39% (0.4–524%)
SG (conventional)	5.8 ± 2.0 (1.8–137)	23% (3–210%)
Osmolality (conventional)	5.8 ± 2.0 (1.6–122)	23% (0–183%)
Creatinine (modified)	6.7 ± 2.0 (1.8–164)	30% (0.3–333%)
SG (modified)	5.9 ± 2.0 (1.7–140)	29% (4–302%)
Osmolality (modified)	5.8 ± 2.0 (1.6–121)	27% (0–249%)

### 3.3 Correction performance comparisons (Criterion A)

**Fig. 2:**
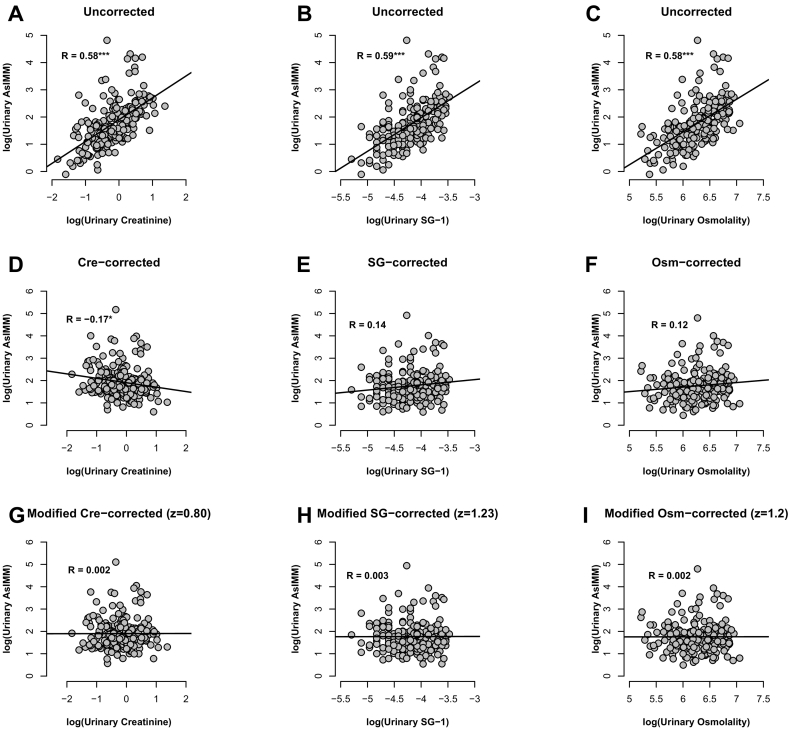
Scatterplots of urinary As^IMM^ against dilution measurements (Criterion A) both pre- (A-C) and post- (D-I) correction by each method investigated. *** and * denote statistical significance to <0.001 and <0.05, respectively.

The performance of the different correction methods relative to both assessment criteria are shown by the Pearson correlations in Table 4. Performances relative to Criterion A are depicted by the scatter plots in Fig. 2. Firstly, correlations of uncorrected urinary As^IMM^ against each of osmolality (*R* = 0.58), SG (*R* = 0.59) and creatinine (R = 0.58) (Fig. 2A–C) demonstrate the substantial amount of dilution driven variation in As^IMM^ concentrations – greater than one third of variation was explained by dilution alone. Conventional correction methods (Fig. 2E and F), with the exception of conventional creatinine (Fig. 2D), removed dilution-derived variation from As^IMM^ concentrations and left no statistically significant correlations between As^IMM^ concentrations and dilution measurements. Conventional creatinine correction resulted in a statistically significant over-correction (*R* = −0.17; 95% CI: −0.30, −0.04) and, while not significant, SG (*R* = 0.14; 95% CI: −0.001, 0.27) and osmolality (*R* = 0.12; 95% CI: −0.02, 0.25) did yield slight under-corrections. Modified corrections, by nature, left near-zero correlations as they were numerically optimized to do so. This is represented graphically in Fig. 1 and demonstrates the potential for improvement of all three methods for this Criterion, especially creatinine correction. In summary, when assessed against Criterion A, the following performance ranking was observed (see Table 4 for significance of differences between non-neighbouring correction methods):

$$\text{Creatinine}\;\left(\text{modified}\right)=\mathrm{SG}\;\left(\text{modified}\right)=\text{Osmolality}\;\left(\text{modified}\right)>\text{Osmolality}\;\left(\text{conventional}\right)\geq \mathrm{SG}\;\left(\text{conventional}\right)>\text{Creatinine}\;\left(\text{conventional}\right)>\text{Uncorrected}$$

**Table 4:** Pearson correlations for performance Criteria A and B across the range of correction methods investigated. Correlations share a letter (a, b and c) when not significantly different from one another. *Uncorrected Criterion A correlations were almost identical for urinary As^IMM^ against each of urinary creatinine, osmolality and specific gravity (see Fig. 2A–C).

Correction method	Criterion A		Criterion B	
	R (95% CI)	Significance	R (95% CI)	Significance
Uncorrected^⁎^	0.58 (0.48, 0.66)		0.65 (0.56, 0.72)	ab
Creatinine (conventional)	−0.17 (−0.30, −0.04)		0.62 (0.53, 0.70)	ac
SG (conventional)	0.14 (−0.001, 0.27)	a	0.68 (0.60, 0.75)	b
Osmolality (conventional)	0.12 (−0.02, 0.25)	a	0.67 (0.58, 0.74)	bc
Creatinine (modified)	0.002 (−0.14, 0.14)	b	0.66 (0.58, 0.73)	b
SG (modified)	0.003 (−0.14, 0.14)	b	0.66 (0.57 0.73)	ab
Osmolality (modified)	0.002 (−0.14, 0.14)	b	0.65 (0.56, 0.72)	ab

### 3.4 Correction performance comparisons (Criterion B)

**Fig. 3:**
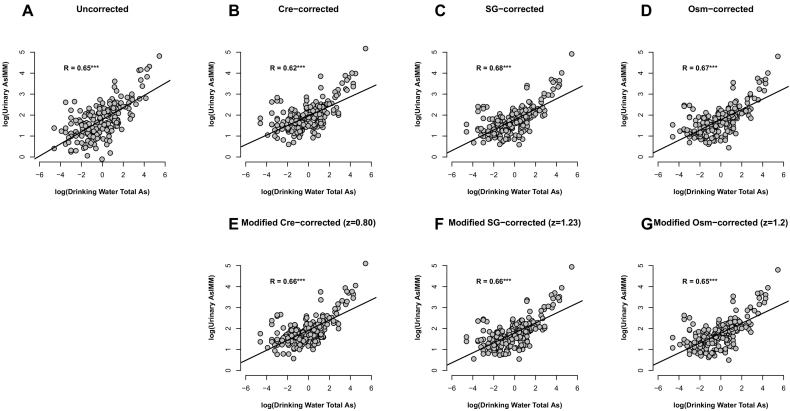
Scatterplots of urinary As^IMM^ against total As in drinking water (Criterion B) both pre- (A) and post- (B-G) correction by each method investigated. *** denotes statistical significance to <0.001.

Correction performances as assessed by Criterion B are also shown by the correlations in Table 4 and the scatterplots in Fig. 3. While not statistically significantly different, correlations of osmolality (both methods), SG (both methods) and modified creatinine corrected As^IMM^ against total As in drinking water were slightly stronger than for uncorrected concentrations. Notably, conventional creatinine adjustment resulted in a weaker correlation than all other, including uncorrected, methods. The following performance ranking was observed relative to Criterion B:

$$\mathrm{SG}\;\left(\text{conventional}\right)\geq \text{Osmolality}\;\left(\text{conventional}\right)\geq \text{Creatinine}\;\left(\text{modified}\right)\geq \mathrm{SG}\;\left(\text{modified}\right)\geq \text{Osmolality}\;\left(\text{modified}\right)\geq \text{Uncorrected}\geq \text{Creatinine}\;\left(\text{conventional}\right)$$

We note that, as can be seen in Fig. 1, there were large discrepancies between the point estimates of *z* coefficients obtained when optimized against either Criterion A or B. For Criterion A versus B: optima were *z* = 0.8 versus 0.49; 1.23 versus 0.72 and 1.2 versus 0.66 for creatinine, SG and osmolality, respectively. Furthermore, for creatinine, improvements for both criteria were achievable relative to conventional adjustment (*z* = 1 - dashed line in Fig. 1), whereas optima for SG and osmolality were at either side of the dashed line, meaning optimization for one Criterion came at the expense of the other. However, differences in Pearson correlations for Criterion B were small between each optimum (e.g. *R* = 0.65 for *z* = 1.2 versus *R* = 0.68 for *z* = 0.66 for osmolality correction) for all three correction methods and only small strength increases in Criterion B correlations were achieved for large differences in *z*. It is noted that the optimum Criterion A *z* coefficients were remarkably similar between SG (*z* = 1.23) and osmolality (*z* = 1.2); slightly more so than for Criterion B optima (0.72 versus 0.66).

## 4 Discussion

We have presented one of few investigations to comparatively assess the performance of urinary dilution correction methods against multiple criteria, including a proposed modification of the mathematical application of three commonly used corrections. Furthermore, this is the first study to compare these methods against a performance criterion based on external environmental exposure – As in drinking water. We observed a substantial (>30%) amount of variation among uncorrected spot As^IMM^ concentrations attributable to dilution. Correction methods had large and variable effects on urinary As^IMM^ concentrations – capable of changing individual concentrations by a factor of five. Conventional SG and osmolality corrections successfully removed significant dilution variation from As^IMM^ concentrations, whereas conventional creatinine over-corrected. It was possible to completely remove dilution variation by modifying all three corrections with *z* coefficients. Similarly, modified creatinine and both conventional and modified applications of specific gravity and osmolality generated stronger correlations of urinary As^IMM^ and drinking water As concentrations than conventional creatinine, which gave weaker correlations than uncorrected values. The performance of correction methods relative to Criterion A was much more susceptible to improvement than Criterion B, relative to which only small differences were observed between methods. Furthermore, creatinine correction showed a much higher potential for modification than SG and osmolality, highlighting the deficiency of creatinine correction in its conventional form in comparison to the two alternative methods.

Our findings are consistent with others, namely our previous investigation (Middleton et al., 2016a), in which we demonstrated that conventional creatinine correction did not perform as well as osmolality in removing dilution variation from urinary lead, cadmium and As^IMM^ concentrations. In the present study we went further an assessed a proposed modification of creatinine, SG and osmolality correction based on previously reported studies (Araki et al., 1990; Vij and Howell, 1998b; Sata and Araki, 1996; Sata et al., 1996) and demonstrated that the efficacy of creatinine correction can be greatly enhanced by a mathematical application which reflects underlying excretion mechanisms; accounting for the discordance between the slope of both creatinine and the analyte being corrected in relation to UFR (Araki et al., 1990). Furthermore the degree of discordance has been shown to be specific to each analyte (Araki et al., 1986a). Our own study is just one of many to address the limitations of creatinine correction, but previous work has tended to focus on the implications of covariates on creatinine excretion (Barr et al., 2005; Nermell et al., 2008b; Gamble and Liu, 2005). We note that such covariates are also present for the alternatives, such as osmolality and SG, now being turned to more frequently (Yeh et al., 2015). For this reason, we recommend taking a more objective approach to comparing and assessing the performance of correction factors.

Our study was limited by a number of factors, including a modest sample size, which likely prevented us from detecting significant differences between many of our performance correlations. The age distribution of study participants, with a mean age of 62 and only 8 individuals under age 40, was also not representative of the wider population and thus did not allow us to perform a systematic analysis of the influence of demographic covariates on correction performance. Our study group was from a rural population of private water supply users (Middleton et al., 2016d), enabling us to assess correction performance against a measure of external As exposure from drinking water. Older participants were more available for participation during daytime sampling visits.

A crude approach was used to derive the *z* coefficient used in modified dilution corrections. Slopes of analyte concentrations against UFR should be derived on single individuals at various hydration states over time and likely vary by demographic and anthropometric factors (Middleton et al., 2016a; Araki et al., 1986b). In the absence of a comprehensive source of empirically derived slopes, a previously employed (Vij and Howell, 1998b) approach was considered a suitable alternative to fulfil the aims of this paper and demonstrate proof of concept. Using this approach, we selected (Fig. 1) *z* values which gave the weakest correlations for corrected analyte concentrations and the dilution metrics used to correct them (Criterion A). We based this selection on the notion that the desired result of correction is to remove dilution variation from the sample set. However, it is likely that a forced correlation of absolute zero (Criterion A optimum) is not completely robust, which may have been evident in the disparity in optima between both criteria. It is possible that the true optima lie somewhere in the middle. Furthermore, the performance extremes for Criterion B (*R* = 0.62-*R* = 0.68) were much more subtle than those for Criterion A and a greater contrast in performance may have been evident if using a more robust urinary exposure biomarker. For example, As^IMM^ incorporates a portion of DMA directly ingested and/or metabolised from dietary As sources and hence not specific to drinking water. Further studies using more specific biomarkers may highlight more obvious performance differences between conventional and modified correction methods. Despite these limitations, the study represents a valid proof of concept with important implications to environmental exposure assessment and the wider discipline of biomonitoring.

Our findings reiterate the widely recognised necessity of dilution correction to remove the large amount of non-exposure relevant variation from spot analyte concentrations. The effect of correction on individual concentrations also supports this, but prompts caution regarding the interpretation of single spot concentrations. For example, in relation to benchmark values such as biomonitoring equivalents (Hays et al., 2010; Hays et al., 2014), and doping tests (Athanasiadou et al., 2018). While spot samples are generally deemed acceptable for biomonitoring applications (Rivera-Núñez et al., 2010), 24 h or longer term sampling regimes are frequently cited as the ideal preferred sampling approach (Cornelis, 1996). However, in addition to being inconvenient and having poor compliance, 24 h samples arguably still require dilution correction if comparing between individuals with highly variable daily water intakes. Therefore, the question of dilution correction remains an important priority in advancing the practice of urinary biomonitoring.

The utility of creatinine in dilution correction has looked bleak for some time, but our results show that an appreciable degree of performance can be enhanced by robust mathematical application – seldom addressed to-date. However, creatinine's performance also depends on its physiological robustness as a proxy of UFR, for which osmolality and SG are superior. Nevertheless, this provides an opportunity for research groups having already obtained data to apply this modified correction method without the need for additional analyses. For example, for studies in which urinary analyte and dilution (most likely creatinine) data were collected, corrections can be re-applied using the modifications outlined in this paper to assess to what extent the findings differ to those made when conventional correction was used. For some analytes, this might be limited to a modest increase in the strength of association between exposure and biomarker concentrations, but it is plausible, for chemicals with very different excretion kinetics relative to dilution markers, that greater influence will be observed. Readers wishing to employ these methods can download R scripts made freely available in our previous publication (Middleton et al., 2016a).

Further research in this area should address the derivation of correction coefficients (*z* values) by generating concentrations slopes for a broad range of analytes against UFR for single individuals over varying states of hydration. This should be conducted on a study group large enough to facilitate analysis of demographic and anthropometric variation. This will enable further optimisation of corrections and could be tailored to specific analytes of interest and population subgroups. In addition, a robust set of assessment criteria for the performance of dilution corrections should be developed, and may differ depending on the intended purpose of the biomonitoring protocol, such as agreement with internal dose measurements.

We have provided further evidence of the need to correct urinary arsenic and other environmental chemical concentrations for dilution variation and, in comparatively assessing a range of alternative methods, concluding that conventional applications are likely sub-optimal as currently employed. Methods can be optimized with a more robust mathematical application, with performance improvement particularly evident for creatinine. Further work is still needed to optimize dilution correction methods with carefully selected criteria to validate their performance and realize the full potential of urine biospecimens in environmental health research and epidemiology.

## Disclaimer

Where authors are identified as personnel of the International Agency for Research on Cancer / World Health Organization, the authors alone are responsible for the views expressed in this article and they do not necessarily represent the decisions, policy or views of the International Agency for Research on Cancer / World Health Organization.
